# Elevated Steady State WBC and Platelet Counts Are Associated with Frequent Emergency Room Use in Adults with Sickle Cell Anemia

**DOI:** 10.1371/journal.pone.0133116

**Published:** 2015-08-06

**Authors:** Susanna A. Curtis, Neeraja Danda, Zipora Etzion, Hillel W. Cohen, Henny H. Billett

**Affiliations:** 1 Division of Hematology, Departments of Medicine and Oncology, Montefiore Medical Center, Bronx, New York, United States of America; 2 Department of Epidemiology and Population Health, Albert Einstein College of Medicine, Bronx, New York, United States of America; Emory University/Georgia Insititute of Technology, UNITED STATES

## Abstract

**Introduction:**

Sickle cell anemia has many sequelae that result in emergency department (ED) use, but a minority of patients with sickle cell disease are frequent utilizers and make up the majority of ED visits. If patients who are likely to be frequent ED can be identified in steady state, they can be treated with disease modifying agents in an attempt to reduce ED use frequency. We sought to identify steady state markers for frequent ED use.

**Methods:**

We identified all patients with SS/Sβ0 seen at our facilities in 2012. Health care utilization over the entire year was calculated and ED visit numbers categorized as either 0–1, 2–5, or 6 or more visits a year. Steady state and acutely active laboratory parameters were collected and analyzed using analysis of variance models and odds ratios.

**Results:**

432 adult sickle cell patients were identified, ages 18–87, 54% female, and 38% had been prescribed hydroxyurea. Of the 432 patients,192 had 0–1 visits in the year, 144 had 2–5 visits in the year, and 96 had >6 visits for a total of 2259 visits. Those who had >6 visits accounted for 1750 (77%) of the total visits for the year. When steady state laboratory markers were examined, each additional 50x109/L platelets was associated with 22% greater risk (p < .001); each 1x109/L of WBC was associated with 11% greater risk (p = .003), and each 1g/dL Hb was associated with 23% lower risk (p = .007) of >6 ED visits/year. We did not observe a relationship between baseline HbF, LDH or reticulocyte count with >6 ED visits.

**Conclusion:**

Patients with elevated white blood cell counts, elevated platelet counts, and low hemoglobin levels exhibited higher risk for frequent ED utilization and could be candidates for early and aggressive therapy with disease modifying agents.

## Introduction

Sickle cell anemia (SCA) is known to have many sequelae that lead to emergency department (ED) utilization, among them cerebral vascular accidents, acute chest syndrome, priapism, and most commonly pain crisis.[1] These admissions come with no small financial cost and detract significantly from the patient’s quality of life.[2–6] However, despite this, many patients with sickle cell disease are able to manage their complications as outpatients, and a minority of patients make up the majority of ED visits.[7–9] If we were able to predict in the clinic who is likely to be a frequent utilizer of the ED, we might be able to use evidenced based management to decide whom to treat with aggressive disease modifying therapy. Hydroxyurea in particular has been shown to be an effective disease modifying agent in reducing emergency department use. [10]

Here we analyze a single center urban cohort of patients with SCA in order to evaluate for steady state markers of frequent emergency department utilization in a single year. We also examine the rate of hydroxyurea prescription and its association with these markers and with high frequency ED use itself. By using steady state biomarkers, it may be possible to personalize therapy before ED use becomes more frequent, with the aim of reducing high frequency ED use and improving quality of life.

## Methods

### Study Population

This study was approved by the Albert Einstein College of Medicine East Campus Institutional Review Board, written informed consent for use of medical records was not obtained however all records were anonymized and de-identified before use. We used our electronic medical records system and Clinical Looking Glass (CLG) to determine the study population. CLG is a user-friendly interactive software application developed at Montefiore Medical Center to evaluate health care quality, effectiveness, and efficiency. The system integrates clinic and administrative data sets allowing clinicians to extract cross-sectional and longitudinal data suitable for epidemiological analyses. We identified patients with only sickle hemoglobin (Hb) who were seen at our institution between 1/1/2012 and 12/31/2012. Sickle Cell Anemia (SCA) was defined as those patients who had a Hb electrophoresis in our system with only Hb S, Hb F, and HbA2 present or those known as SCA but whose recent transfusion accounted for any Hb A seen. Patients with HbSC and HbSß^+^ thalassemia were excluded as these syndromes have been shown to have differing levels of steady state laboratory markers and of health care utilization.[5] General demographic data were obtained by CLG and identified medical charts were reviewed.

### Laboratory Determinants

Hb electrophoresis was measured by high performance liquid chromatography. All “steady state” and “active” results of each of the following routine laboratory assessments obtained from 1/1/2012 until 12/31/2012 were obtained. Steady state laboratory tests were defined as those not within a day of an ED visit or a week of a hospital admission. “Active” state laboratory tests, defined as those within one day of an ED visit, were averaged separately. If more than one assessment was available, the repeated results were averaged: Hb, red blood cells (RBC), hematocrit (Hct), mean cell volume (MCV), mean cell hemoglobin concentration (MCHC), absolute reticulocyte count (retic), platelets (Plt), white blood cell count (WBC), serum creatinine (Cr), albumin (Alb), total protein (Tprot), alkaline phosphatase, serum alanine transaminase, serum aspartate aminotransferase (AlkPhos, ALT, AST respectively), total bilirubin and direct bilirubin, (TB, DB respectively), lactate dehydrogenase (LDH), and percent HbF were obtained. HbF, age, and weight were not separated by “steady state” or “active” status.

### Clinical Data

The total number of emergency department visits, inpatient admissions, and clinic visits was recorded for each patient within 1/1/2012 to 12/31/2012. Hydroxyurea use was defined as positive if the patient was given one or more prescriptions for hydroxyurea within 1/1/2012 until 12/31/2012. The first script of Hydroxyurea dose that year was obtained. Age was defined as age at January 1^st^, 2012. Weight was defined as the first recorded weight measurement from 1/1/2012 until 12/31/2012.

### Statistical Analysis

Data were entered into Excel spreadsheets. (Microsoft Excel, Microsoft Corp., Redmond, WA). Statistical analyses were performed with SPSS for Windows (Version 20, SPSS, Inc., Chicago, IL)). Data were analyzed for normality; parametric values were reported as mean ±SD, non-parametric values were reported as medians and interquartile ranges. Parametric and non-parametric bivariate tests of association were used as appropriate. Emergency department visits were categorized by number of visits as 0–1, 2–5, and ≥6. Continuous parametric variables were compared over these categories using ANOVA while non-parametric variables were compared using the Kruskal-Wallis test. Each measure of interest was tested for association with the number of visits separately. Associations of continuous variables with number of visits were also assessed with Pearson (linear) or Spearman (monotonic) correlations as appropriate. The odds ratios and 95% CI of ≥6 ED visits a year for each independent variable were estimated with binary logistic regression models adjusted for age. A two-tailed alpha of .05 was used to denote statistical significance. Logistic regression analyses were used to model algorithms for ED use.

## Results

### Demographic Data

The initial decision to categorize ED utilization was made a priori; patients were categorized into groups of 0, 1, 2–5, or ≥6 visits per year. However, review of the data on cohorts 0 visits and 1 visit per year were statistically equivalent and their data were combined. 432 adult sickle cell patients were identified; their ages ranged from 18–87 yrs and 54% were female. 192 patients had 0–1 ED visits within the year, they were 50% female and had a mean age of 34.9 ±13 years; 144 had 2–5 visits in the year, they were 58% female and had a mean age of 34.4 ±13 years; 96 had ≥6 visits for a total of 2259 visits, they were 55% female and had a mean age of 29.7 ±8.3 years. Patients who had ≥6 visits were <25% of the sample but accounted for 1750 (77%) of the total visits for the year. Age was significantly different between cohorts (p = .002) but gender was not significantly different (p = 0.4). 38.1% of the patients had been given a prescription for hydroxyurea within the year. Increased ED frequency was also significantly associated with increasing inpatient admissions, the 0–1 ED visits category had a median of 0 admissions a year while the ≥6 category had an average of 5 admissions a year (p < .001).

### Steady State Laboratory Determinants

Of the initial 432 patients, 311 had complete laboratory values. When laboratory determinants were examined, increased emergency department utilization was significantly and linearly associated with elevated steady state WBC (p<0.001), elevated steady state platelet count (p<0.001), lower steady state RBC (p<0.001), lower steady state Hb (p < .001) and lower steady state albumin (p = .02, Table 1). Table 2 shows the odds ratios (95% CI), adjusted for age, for high ED utilization (≥6 emergency room visits) with each steady state laboratory parameter. For each unit increase of 50 x10^9^/L in platelet count, there was an associated 22% greater risk of high ED utilization, p<0.001. For each 1 x10^9^/L increase in WBC, there was an associated 11% greater odds of ≥6 ED visits/year, p = 0.003, while each unit of 1 x10^9^/L RBC and 1g/dl Hb was associated with a 49% and 23% reduced odds of high ED utilization (p = .006, p = .007 respectively). For each 1g/dl of albumin there was a 60% lower risk of frequent ED use (p = .01, Table 2). While LDH and Cr were significantly different among categories of ED use, the associations were not linear.

**Table 1:** Steady state laboratory determinants for each ED utilization cohort. Normalized data reported as means with SDs, non-normalized data reported as medians with 25% and 75%. P values are for ANOVAs done over all 3 categories.

	Steady State Data			
ED Visits	0–1	2–5	≥6	p
n	192	144	96	
**RBC (x10^12/L)**	3.0±0.8	2.7±0.6	2.6±0.5	**< .001**
**Hb (g/dL)**	8.8±1.7	8.1±1.4	8.1±1.3	**< .001**
**MCV (g/dL)**	90.2±13.6	90.6±11.7	92.0±11.0	0.6
**MCHC (g/dL)**	34.1±1.3	34.3±1.2	34.0±1.7	0.3
**Reticulocytes (x10^9/L)**	151.7(219.8/330.8)	271.3(172.0/362.9)	247.4(180.8/342.0)	0.1
**Platelet (x10^9/L)**	337.8±127.7	355.8±110.2	428.2±150.8	**< .001**
**WBC (x10^9/L)**	9.5±4.1	10.9±12.0	12.0±4.1	**< .001**
**LDH (U/L)**	351.0(282.0/458.5)	429.3(329.0/555.0)	397.0(303.8/540.0)	**0.02**
**Cr (mg/dL)**	0.7(0.6/0.9)	0.6(0.5/0.8)	0.7(0.6/0.8)	**0.02**
**Alb (g/dL)**	4.4±0.4	4.32±0.4	4.26±0.4	**0.02**
**Tprot (g/dL)**	7.7±0.7	7.7±0.6	7.5±0.7	0.1
**AlkPhos (U/L)**	83.8(65.9/116.8)	91.3(73.1/129.5)	101.8(70.8/138.7)	0.08
**AST (U/L)**	33.8(25.7/48.8)	38.0(31.5/52.0)	39.5(30.4/55.9)	**0.005**
**ALT (U/L)**	21.4(16.0/32.1)	20.7(16.0/30.0)	22.8(15.9/33.2)	0.6
**Tbili (mg/dL)**	2.3(1.4/3.8)	2.6(1.8/4.3)	2.3(1.5/4.0)	0.1
**Dbili (mg/dL)**	0.4(0.3/0.5)	0.4(0.3/0.5)	0.4(0.3/0.6)	0.05
**Weight (KG)**	68.2±13.7	66.0±14.9	66.6±13.6	0.4
**HbF (%)**	5.3(3.0/13.0)	6.3(3.7/9.9)	6.4 (3.8/11.2)	1.0
**Hydroxyurea (% with script)**	23	40.3	65.3	**< .001**
**Dose (mg/kg)**	16.6 (7.9/24.8)	17.8 (11.6/23.1)	19.7 (10.7/27.3)	0.4
**Admissions (/year)**	0 (0/1)	2 (1/3)	5 (3/8)	**< .001**

**Table 2:** Age adjusted odds ratios (OR, 95%CI) for steady state laboratory determinants for those with ≥6 ED visits.

Parameter	Odds Ratio	p
**RBC (per 1 x10^12/L)**	0.51 (0.32–0.82)	**0.006**
**Hb (per 1 g/dl)**	0.77 (0.63–0.93)	**0.007**
**MCV (per 1g/dL)**	1.02 (0.99–1.04)	0.2
**MCHC (per 1g/dL)**	0.89 (0.74–1.07)	0.2
**Retic (per 1x10^9/L)**	1.00 (1.00–1.00)	1.0b
**Platelet (per 50x10^9/L)**	1.22 (1.10–1.36)	**< .001**
**WBC (per 1x10^9/L)**	1.11 (1.04–1.20)	**0.003**
**LDH (per 1 U/L)**	1.02 (0.87–1.20)	0.8
**Cr (per 1 mg/dL)**	1.11 (0.90–1.37)	0.3
**Alb (per 1 g/dL)**	0.40 (0.20–0.80)	**0.01**
**Tprot (per 1 g/dL)**	0.60 (0.37–0.97)	**0.04**
**AlkPhos (per 1 U/L)**	1.32 (1.00–1.74)	**0.05**
**AST (per 1 U/L)**	1.01 (1.00–1.02)	0.1
**ALT (per 1 U/L)**	1.01 (1.00–1.02)	0.08
**Tbili (per 1 mg/dL)**	1.00 (0.90–1.11)	1.0
**Dbili (per 1 mg/dL)**	1.23 (0.88–1.71)	0.2
**Weight (per 1 KG)**	1.00 (0.98–1.02)	1.0
**HbF (per 1%)**	1.01 (0.78–1.06)	0.8
**Hydroxyurea (with script)**	3.96 (2.43–6.44)	**< .001**
**Dose (per 1 gm)**	1.00 (1.00–1.00)	0.9

We developed an algorithm which would differentiate those whose ED utilization was not ≥6 visits/year. Using the average Hb for the 0–5 utilizers (8.4g/dl), median WBC (9.5 x10^9^/L), and median platelet count (341 x10^9^/L), we assigned 1 point each for WBC >9 x10^9^/L and platelet count 300 x10^9^/L and 1 point for Hb levels <9g/dl. 78 of the 311 (25%) patients incorporated in the model had a score of 0 or 1; of those 78 low scoring patients, 69 were lower ED utilizers. This score was found to be 34% sensitive and 89% specific for lower ED utilization in our cohort.

### Active State Determinants

When active parameters were compared to steady state parameters, laboratory values among the ED categories overlapped and there were no significant differences in the levels of anemia or the degree of leukocytosis; *all* categories showed leukocytosis and decreased Hb levels while presenting acutely (Table 3). High platelet counts, however, remained different among ED utilization categories and were again associated with more frequent ED visits (p = .02). In contrast to the association of increased ED utilization with decreasing albumin levels in steady state, in the active state higher albumin levels, perhaps reflecting dehydration, were associated with a higher ED use category (p = .02). As in steady state, creatinine levels were significantly different in each category but no linear association was demonstrated.

**Table 3:** Laboratory parameters during steady state and during “active” (1 day within ED visit or 1 week within a hospital admission) state. Normalized data reported as means with SDs, non-normalized data reported as medians with 25% and 75% P values are for ANOVAs done over all 3 categories.

ED Visits	0–1		2–5		≥6		p for active
n	192		144		96		variable
	Steady State	Active	Steady State	Active	Steady State	Active	
**RBC (x10^12/L)**	3.0±0.8	2.8±0.7	2.7±.6	2.7±0.7	2.6±0.5	2.6±0.6	0.2
**Hb (g/dL)**	8.8±1.7	8.3±1.6	8.1±1.4	8.0±1.4	8.1±1.3	8.0±1.2	0.3
**MCV (g/dL)**	90.2±13.6	88.4±13.0	90.6±11.7	88.0±12.1	92.0±11.0	89.3±12.8	0.8
**MCHC (g/dL)**	34.1±1.3	34.4±1.3	34.3±1.2	34.5±1.4	34.0±1.7	34.4±1.1	0.8
**Retic (x10^9/L)**	219.8 (151.7/330.8)	264.3(169.0/376.4)	271.3(172.0/362.9)	299.8(197.4/405.9)	247.4(180.8/342.0)	278.6(220.3/377.4)	0.3
**Platelet (x10^9/L)**	337.8±127.7	338.6±111.96	355.8±110.2	386.1±131.0	428.2+150.8	402.3±106.9	**0.02**
**WBC (x10^9/L)**	9.5±4.1	13.4±6.2	10.9±12.0	13.2±4.4	12.0±4.1	13.8±3.9	0.7
**LDH (U/L)**	351.0(282.0/458.5)	484.0(281.5/602.9)	429.3(329.0/555.0)	484.0(382.3/637.0)	397.0(303.8/540.0)	426.0(341.8/563.5)	0.3
**Cr (mg/dL)**	0.7(0.6/0.9)	0.8(0.6/1.1)	0.6(0.5/0.8)	0.7(0.5/0.9)	0.7(0.6/0.8)	0.7(0.6/0.8)	**0.006**
**Alb (g/dL)**	4.4±0.4	4.2±0.5	4.32±0.4	4.3±0.4	4.26±0.4	4.4±0.4	**0.02**
**Tprot (g/dL)**	7.7±0.7	7.4±0.7	7.7±0.6	7.6±0.6	7.5±0.7	7.6±0.5	0.2
**AlkPhos (U/L)**	83.8(65.9/116.8)	90.0(73.5/172.5)	91.3(73.1/129.5)	84.7(68.3/115.5)	101.8(70.8/138.7)	94.6(75.1/121.0)	0.2
**AST (U/L)**	33.8(25.7/48.8)	45.5(28.8/63.1)	38.0(31.5/52.0)	44.5(32.9/58.3)	39.5(30.4/55.9)	43.2(34.1/56.7)	1
**ALT (U/L)**	21.4(16.0/32.1)	25.5(16.0/38.5)	20.7(16.0/30.0)	20.4(15.5/33.0)	22.8(15.9/33.2)	22.9(17.1/33.3)	0.5
**Tbili (mg/dL)**	2.3(1.4/3.8)	3.0(1.4/5.3)	2.6(1.8/4.3)	2.9(1.9/4.5)	2.3(1.5/4.0)	2.3(1.7/3.9)	0.5
**Dbili (mg/dL)**	0.4(0.3/0.5)	0.5(0.3/0.8)	0.4(0.3/0.5)	0.4(0.3/0.6)	0.4(0.3/0.6)	0.4(0.3/0.5)	0.1

### Steady State Laboratory Determinants by Hydroxyurea Script

Hydroxyurea use was examined as previous studies have shown that it affects both frequency of ED use and steady state laboratory parameters themselves. [11] The cohort prescribed hydroxyurea had significantly higher MCVs than the cohort not prescribed hydroxyurea across all 3 categories of ED use, suggesting that they were taking the medication. Both the hydroxyurea and the non-hydroxyurea cohorts retained linear and significant associations of higher WBC and platelets counts with greater ED use (Table 4). Though increasing anemia was still linearly associated with higher ED use in the hydroxyurea group, in the nonhydroxyurea group the association was no longer strictly linear.

**Table 4:** Steady state laboratory determinants for each ED utilization cohort according to hydroxyurea use. Normalized data reported as means with SDs, non-normalized data reported as medians with 25% and 75% P values are for ANOVAs done over all 3 categories.

	No Hydroxyurea				Hydroxyurea			
	0–1	2–5	≥6	p	0–1	2–5	≥6	p
**RBC (x10^12/L)**	3.0±0.9	2.6±0.7	2.7±0.5	**0.009**	2.8±0.5	2.7±0.6	2.6±.5	0.4
**Hb (g/dL)**	8.7±1.8	7.8±1.4	7.9±1.6	**0.001**	9.0±1.5	8.5±1.4	8.2±1.4	**0.02**
**MCV (g/dL)**	87.3±13.3	88.0±10.6	86.8±9.2	0.9	96.8±12.0	93.3±12.4	94.3±11.0	0.4
**MCHC (g/dL)**	34.1±1.4	34.1±1.3	34.1±0.9	0.9	34.3±1.1	34.5±1.2	33.9±2.0	0.2
**Retic (x10^9/L)**	228.0(162.0/337.0)	279.0(178.3/415.0)	291.0(183.0/328.0)	0.2	199.3(91.2/325.4)	266.0(168.2.0/351.0)	229.3(166.4/342.0)	0.3
**Platelet (x10^9/L)**	334.3±125.7	348.1±123.0	451.0±151.0	**0.001**	337.8±125.4	363.9±95.8	420.3±152.0	**0.006**
**WBC (x10^9/L)**	9.5±3.4	11.5±3.5	13.2±3.6	**< .001**	9.1±4.6	10.3±3.1	11.5±4.2	**0.02**
**LDH (U/L)**	340.5(268.7/469.0)	484.0(329.0/593.0)	421.4(281.5/558.3)	**0.03**	374.5(322.6/439.0)	422.5(328.8/547.7)	372.0(303.2/569.3)	0.5
**Cr (mg/dL)**	.8(.6/.9)	.6(.5/.8)	.8(.6/1.0)	**0.05**	.7(.6/.9)	.7(.5/.8)	.7(.6/.8)	0.08
**Alb (g/dL)**	4.4±0.4	4.3±0.4	4.2±0.3	0.07	4.5±0.4	4.4±0.4	4.3±0.6	0.2
**Tprot (g/dL)**	7.7±0.7	7.5±0.7	7.6±0.6	0.3	7.7±0.5	7.8±0.6	7.4±0.5	**0.002**
**AlkPhos (U/L)**	82.5(65.0/112.8)	89.5(67.8/131.5)	98.5(75.7/145.2)	0.2	91.0(71.0/121.8)	94.7(73.6/129.8)	104.5(69.4/133.2)	0.7
**AST (U/L)**	33.8(25.3/53.0)	37.8(32.0/53.0)	38.3(28.1/61.9)	0.3	34.7(27.0/42.0)	38.3(29.9/51.7)	41.9(31.4/52.8)	0.1
**ALT (U/L)**	20.6(16.0/31.0)	22.0(16.0/33.8)	19.0(14.2/29.5)	0.6	23.3(17.0/35.3)	19.8(15.8/28.4)	24.0(16.6/45.1)	0.1
**Tbili (mg/dL)**	2.3(1.5/3.9)	2.8(1.8/4.3)	2.4(1.7/3.7)	0.3	2.6(1.2/3.9)	2.5(1.8/4.1)	2.3(1.4/4.1)	0.6
**Dbili (mg/dL)**	.4(.3/.5)	.5(.4/.6)	.4(.3/.8)	**0.02**	0.3(0.3/0.4)	.4(.3/.5)	.4(.3/.6)	0.4
**Weight (KG)**	67.3±14.0	64.9±14.1	69.7±13.2	0.3	70.5±12.7	67.3±15.8	65.2±13.7	0.2
**HbF (%)**	5.6(2.9/12.5)	5.8(2.8/8.2)	7.9(4.9/12.6)	0.3	7.1(3.6/13.8)	8.2(4.2/14.4)	5.2(2.8/11.4)	0.4

We were unable to show that hydroxyurea prescription frequency was associated with less ED use. While 23% of those who were seen in the ED 0–1 times a year were prescribed hydroxyurea, 40.3% of those who in the 2–5 times cohort and 65.3% of those who had ≥6 ED visits/year (p < .001) were prescribed hydroxyurea. The average first dose prescribed that year did not significantly differ between the three groups (16.6 mg/kg, 17.8 mg/kg, and 19.7 mg/kg in the 0–1, 2–5 and ≥6 groups respectively, p = 0.40) The ≥6 ED visits cohort, despite the increased MCV and lower WBC, did not show a more elevated HbF level than the non-hydroxyurea cohort.

### Steady State Laboratory Determinants by Age

ED visit categories were also examined by age groups as prior studies have shown that laboratory determinants differ with age in patients with SCA. [12] When categories were examined by those <40 years and those ≥40 years old, again platelets and WBC remained significantly associated with ED use in both groups, although the older cohort had lower means at each level (Table 5). RBC, Hb, and albumin remained significantly linearly associated with ED use in the younger cohorts but not in the older cohorts.

**Table 5:** Steady state laboratory determinants for each ED utilization for younger (<40yrs) and older (≥40yrs) cohorts. Normalized data reported as means with SDs, non-normalized data reported as medians with 25% and 75% P values are for ANOVAs done over all 3 categories.

	Age<40				Age>40			
	0–1	2–5	6+	P	0–1	2–5	6+	P
**RBC (x10^6/uL)**	3.0±0.8	2.8±0.6	2.6±0.5	**0.001**	2.9±0.9	2.5±0.6	2.7±0.6	0.1
**Hb (g/dL)**	9.0±1.7	8.4±1.3	8.0±1.3	**< .001**	8.6±0.6	7.7±1.5	8.2±1.3	0.08
**MCV (g/dL)**	90.0±13.1	88.0±11.3	91.7±11.2	0.2	90.3±14.6	92.8±11.6	92.3±12.8	0.6
**MCHC (g/dL)**	34.3±1.2	34.3±1.2	34.1±4.4	0.3	33.7±1.3	34.2±1.3	33.8±0.9	0.2
**Retic (x10^9/L)**	260 (167.5/346)	271.3(178.5/362.9)	247.4(180.8/341.2)	0.8	192.4(138.9/262.8)	270.3(171.9/347.5)	230.4(178.8/291.1)	0.2
**Platelet (x10^9/L)**	355.8±138.1	382.6±109.7	429.8±157.8	**0.01**	294.2±91.9	309.3±95.8	420.2+113.4	**0.001**
**WBC (x10^9/L)**	9.9±3.9	11.5±3.4	12.1±4.1	**0.002**	8.1±3.2	9.9±3.0	11.9±4.2	**0.003**
**LDH (U/L)**	360.8(284.3/467.8)	420.0(329.0/543.7)	429.0(322.0/579.0)	0.1	398.0(344.5/538.5)	337.9(295.0/477.5)	554.0(322.6/610.8)	0.4
**Cr (mg/dL)**	0.7(0.6/0.8)	0.6(0.5/0.7)	0.7(0.6/0.8)	**0.005**	0.9(08/1.3)	0.8(0.6/1.2)	0.7(0.6/1.0)	0.1
**Alb (g/dL)**	4.5±0.4	4.4±0.4	4.3±0.4	**0.005**	4.4±0.5	4.2±0.5	4.3±0.3	0.1
**Tprot (g/dL)**	7.7±0.6	7.6±0.5	7.5±0.5	**0.005**	7.5±0.6	7.8±0.7	7.5±0.5	0.5
**AlkPhos (U/L)**	83.7(65.0/112.4)	88.8(71.0/113.1)	99.0(70.0/133.1)	0.3	67.0(84.5/121.1)	112.3(82.3/145.0)	108.5(86.7/140.5)	0.2
**AST (U/L)**	35.7(28.0/48.6)	38.3(30.6/53.2)	39.5(31.0/56.8)	0.2	37.0(25.3/41.9)	36.0(26.2/56.8)	46.7(29.0/58.4)	0.3
**ALT (U/L)**	22.0(17.0/30.0)	22.0(15.7/33.3)	20.5(15.9/31.0)	0.4	19.0(14.2/26.8)	20.5(15.5/31.8)	32.1(22.5/30.8)	0.6
**Tbili (mg/dL)**	2.1(1.5/3.9)	2.8(1.9/4.1)	2.6(1.6/4.0)	0.5	2.0(1.3/3.8)	2.5(1.4/4.0)	3.1(2.3/5.9)	0.1
**Dbili (mg/dL)**	0.4(0.3/0.5)	0.5(0.3/0.6)	0.4(0.3/0.5)	0.2	0.4(0.3/0.4)	0.4(0.3/0.5)	0.5(0.4/0.7)	0.2
**Weight (kg)**	67.5±14.2	65.9±14.9	66.6±14.1	0.6	69.5±12.5	66.2±15.0	66.7±11.5	0.4
**HbF (%)**	6.9(3.0/13.0)	6.2(3.6/9.5)	5.9(3.8/10.8)	0.9	5.1(3.3/13.1)	7.7(4.4/12.1)	5.1(9.5/13.8)	0.7

## Discussion

Among patients with sickle cell anemia a relatively small percent of the population accounts for the majority of emergency department utilization. Previous studies have demonstrated that there may be some variability in which patients will be high utilizers from year to year and who will be among these high frequency utilizers may be harder to predict in an emergent setting.[9,13,14] It has been shown that it is difficult to identify objective parameters for sickle cell crisis, although there is some evidence for an association of crisis with increasing LDH levels and a decrease in dense cells, as demonstrated by decreasing RDW, indirect bilirubin and MCV.[15–17] However, the ability to predict which patients will be high utilizers in a non-emergent setting could allow for early induction of disease modifying therapy with the aim of reducing future emergency department use. In our study we showed that those who have more frequent ED visits a year also have more annual admissions, suggesting that reducing emergency department use may reduce hospital admission rates as well.

We demonstrate that when patients present to the ED, it is more difficult to differentiate those who are less frequent utilizers of the ED from those who are more frequent utilizers, since the typical monitored laboratory values become more similar. However when steady state values were examined, frequent utilizers had significantly higher white blood cell counts, platelet counts, and they were significantly more anemic. The high WBC and platelet counts suggest that frequent ED utilizers are in an inflammatory state even at their baseline steady state. These increases in WBC and platelet levels remain correlated with frequent ED use in patients prescibed hydroxyurea and non-hydroxyurea and in both older and younger cohorts of patients. Higher levels of inflammatory markers have been associated with more frequent sickle cell crisis, pulmonary hypertension, sickle cell retinopathy, acute chest syndrome, and stroke. [18–21] Studies have shown that anemia and elevations of WBC as well as elevated of VCAM and ICAM, two molecules associated with adhesion of leukocytes to endothelium, are markers for complications, including ACS, crisis, and early mortality. [22–27] Our data are the first to observe that WBC is also a marker for frequent ED utilization. Our model shows that those who will not be high utilizers often do not have elevated markers and a score of ≤1 on our model was 89% specific for low or medium utilization.

Frequent ED utilizers also have higher AST, a marker for hemolysis in the setting of otherwise normal liver tests and alkaline phosphatate. However other markers of hemolysis, reticulocyte count, LDH, indirect bilirubin, did not show a linear association with admissions suggesting that AST may not be a marker for hemolysis in this case. As AST and alkaline phosphatase are found in multiple organ systems in the body they may instead be markers for other underlying pathologies, particularly liver or bone marrow.

These results suggest that those patients with SCA who exhibit baseline leukocytosis and thrombocytosis could be candidates for more aggressive therapy with disease modifying agents. Hydroxyurea has been shown to reduce levels of white blood cells, platelets, and intracellular adhesion molecules and to result in fewer episodes of pain crisis as well as lower overall emergency room utilization.[10,11,26,27] Previous studies have shown that frequent ED utilizers are more likely to be prescribed hydroxyurea, likely due to their increased presentation to the medical system, and that hydroxyurea is an effective disease modifying agent in reducing emergency room use relative to the patients’ own previous rate of use.[10,27] In this study, frequent ED utilizers were 2.8 times more likely to be prescribed hydroxyurea, consistent with the previous studies, though we lack data from their pre-HU course to assess if their ED use was relatively decreased when compared to their previous use. Thus, though our frequent ED visit patients are prescribed hydroxyurea more often, this does not suggest that hydroxyurea is not reducing ED use as they may be visiting the ED less frequently than they would have were they not prescribed the medication. An interesting observation is that the group on hydroxyurea with ≥6ED visits had higher Hb levels, high MCVs, lower WBC than those not on hydroxyurea. This suggests that these high ED utilizers were adherent with the medication but still had low HbF levels. Our data did not demonstrate that HbF level was a significant factor in ED utilization frequency. We could not detect a difference in dosing between the hydroxyurea cohorts, and the lack of a beneficial HbF effect be due to under-dosing. A study in 1995 showed that, in a cohort of 303 patients after 6 months of hydroxyurea treatment, only 33% of patients had achieved maximal tolerated doses. [27] Although current recommendations suggest that, in the absence of intolerable side effects, doses should be titrated upwards until mild myelosuppression is achieved (absolute neutrophil count 2–4 x10^9^/L), this is not commonly attempted. [4,28] Another possibility is that these may represent “nonresponders” since the MCV and the WBC did behave in a hydroxyurea-appropriate manner. We suggest that frequent ED utilizers might benefit from early hydroxyurea use, and particularly that they should have maximal titration of dosing with goals of relative leukopenia and thrombocytopenia, in conjunction with any novel therapies that may be developed.

Our study has several limitations; the most serious is that it is a retrospective cohort study of a single year. Frequency of ED use can change in a patient, so it might be important to observe whether long term variations in steady state laboratory parameters correlate with long term variations in ED use. Secondly, we did not record actual hydroxyurea use over the year but instead used the first script of the year as the steady state dose. Thirdly, we did not adjust for transfusions. Lastly, we do not have accurate data on the reason for the ED presentation since the most common ED diagnosis was just “sickle cell disease” without any further coding suggesting an acute etiology. We therefore made a decision to use ED presentation alone as the important outcome.

In conclusion, our data suggest that elevated steady state WBC and platelet levels are markers for increased emergency room utilization and may serve as indications to maximize therapy for this subpopulation. We have develop a simple model that may be used to differentiate those who are less likely to require aggressive therapy from those who may suffer a potentially more volatile course.

## Supporting Information

S1 File The original laboratory and clinical data has been attached as an excel spreadsheet. (XLS)
